# Therapeutic Potential of *Ficus benjamina*: Phytochemical Identification and Investigation of Antimicrobial, Anticancer, Pro-Wound-Healing, and Anti-Inflammatory Properties

**DOI:** 10.3390/molecules30091961

**Published:** 2025-04-28

**Authors:** Arik Dahan, Ludmila Yarmolinsky, Arie Budovsky, Boris Khalfin, Shimon Ben-Shabat

**Affiliations:** 1 Department of Clinical Pharmacology, Faculty of Health Sciences, Ben-Gurion University of the Negev, Beer-Sheva 8410501, Israel; yludmila@post.bgu.ac.il (L.Y.); boriskh83@gmail.com (B.K.); 2 Research & Development Authority, Barzilai University Medical Center, Ashkelon 7830604, Israel; abudovsky@gmail.com

**Keywords:** *Ficus benjamina*, phytochemicals, antimicrobial activity, biofilm, anticancer, wound healing, anti-inflammatory, bioinformatics

## Abstract

*Ficus benjamina* is a common park tree, with previous reports of some medicinal properties. In this work, we identified and explored phytochemicals from *F. benjamina* for potential antimicrobial, pro-wound-healing, anti-inflammatory, and effect on cancer cell lines’ proliferation, both experimentally and bioinformatically. Gas chromatography/mass spectrometry (GC/MS) analysis was performed to identify the volatile compounds. The nonvolatile active components of the extract were identified by HPLC and LC-ESI-MS. We found that some drug-resistant microorganisms (*Escherichia coli*, *Klebsiella pneumoniae*, *Acinetobacter baumannii*, *Serratia marcescens*, and *Salmonella enteritidis*) were inhibited by the extract, the 80% fraction, and all the identified flavonoids except quercetin 3-*O*-rutinoside. Furthermore, the extract and above-mentioned compound also inhibited the growth of biofilm-producing bacterium. The extract and 80% fraction were very potent (*p* < 0.001) at inducing death of MCF7 and U87 cancer cell cultures and were more effective in that than the chemotherapeutic agent doxorubicin which served as a positive control. Additionally, the extract of *F. benjamina*, the 80% fraction, and selected phytochemicals had pronounced pro-wound-healing properties. Finally, the extracts, the 80% fraction, caffeic acid, kaempferol 3-*O*-rutinoside, and kaempferol 3-*O*-robinobioside significantly inhibited the secretion of pro-inflammatory cytokines, IL-6 and IL-8 (*p* < 0.001). In conclusion, this comprehensive research revealed convincing and promising indications of significant therapeutic potential of a *F. benjamina* extract and its active phytochemicals.

## 1. Introduction

The weeping fig (*Ficus benjamina*) is a well-known park tree in tropical countries and a decorative indoor plant that belongs to the *Moraceae* family [1]. Some medicinal properties of *F. benjamina* have been reported, including anticancer [2], antioxidant [3], antiviral [1,4], and antimicrobial [3] effects; however, inclusive analysis of its therapeutic potential cannot be found in the literature. Hence, this plant and its phytochemicals clearly deserve a much more thorough and comprehensive investigation. In support of this notion are the significant antioxidant properties of *F. benjamina*, as oxidative stress is a critical mechanism in the onset and progression of many diseases [5], including infections [6], inflammations [7], cancer [8,9], and others. We hypothesized that the extract of *F. benjamina* may have antimicrobial, pro-wound-healing, anti-inflammatory, and anticancer properties. Hence, we chose to study the potential effects of *F. benjamina* on all of these important pharmacological actions.

Although antimicrobial properties of *F. benjamina* have been reported [3], testing of this plant and its compounds against drug-resistant microorganisms and microorganisms capable of forming biofilms cannot be found in the literature. According to the World Health Organization, there are two major urgent problems in modern medicine: bacterial resistance to antibiotics and resistance to chemotherapy [10]. In particular, drug-resistant microorganisms represent a severe threat to public health. Due to the COVID-19 pandemic, an increase in antimicrobial resistance was observed [11]. Even a higher threat is posed by biofilms as biofilm-forming microorganisms acquire, very rapidly, an increased resistance to antibiotics, high infectivity, and strong viability [12]. The following modes of action lay the foundation of this phenomenon: slow penetration of the antibiotics into the biofilm’s extracellular matrix, decreased growth rate of bacteria within the biofilm, and various changes as a result of the interactions between the microorganisms with the biofilm [13]. Thus, research aimed at identifying new chemical entities possessing such pharmacological activities is a very important strategy to tackle this urgent problem.

Nowadays, cancer treatment is multi-modal and typically consists of surgery, chemotherapy, targeted therapy, immunotherapy, and radiotherapy [14]. In particular, chemotherapeutic agents, in many cases, may indiscriminately attack both cancer and healthy cells [15]. It was reported that patients who underwent chemotherapy are at increased risk for developing other types of cancer [16]. For example, doxorubicin (DOX) is an important chemotherapeutic drug that significantly improves survival of oncology patients with various tumors [17]. However, the majority of studies demonstrated the harmful effects of this drug on ovarian function [18], the heart (cardiomyopathy, arrhythmia, valve injury, myocarditis, pericarditis, cardiac insufficiency, and myocardial ischemia) [19], brain, kidney, and liver [19]. Another important challenges of modern medicine are poor wound healing (WH) and inflammation [20]. Unfortunately, no pharmacological treatments able to enhance WH and reduce inflammation without potential side effects are available.

Given that *F. benjamina* and its phytochemical constituents may be effective for all of the above-mentioned medical challenges, we have identified and investigated the major phytochemical constituents, using bioinformatics and experimental methods, to find their effect on cancer cell line proliferation and antimicrobial, pro-wound-healing, and anti-inflammatory properties. Hence, this work has the potential to identify new phytochemical entities possessing important pharmacological activities, aiming to tackle some of the most urgent medical challenges of our times.

## 2. Results

### 2.1. Identification of Phytochemicals

The ethanolic leave extract of *F. benjamina* was fractionated. Among the obtained fractions, only the 80% fraction was active in all the performed experiments, and so our research continued and focused on this fraction. In addition, some volatile phytochemicals were found in the plant leaves. Based on the thorough chemical analysis, the phytochemicals identified in the leaves of *F. benjamina* are summarized in Table 1, alongside their concentrations in the extract. The pure compounds identified in the leaves were purchased for further experiments.

### 2.2. Antimicrobial Activity

**Figure 1:**
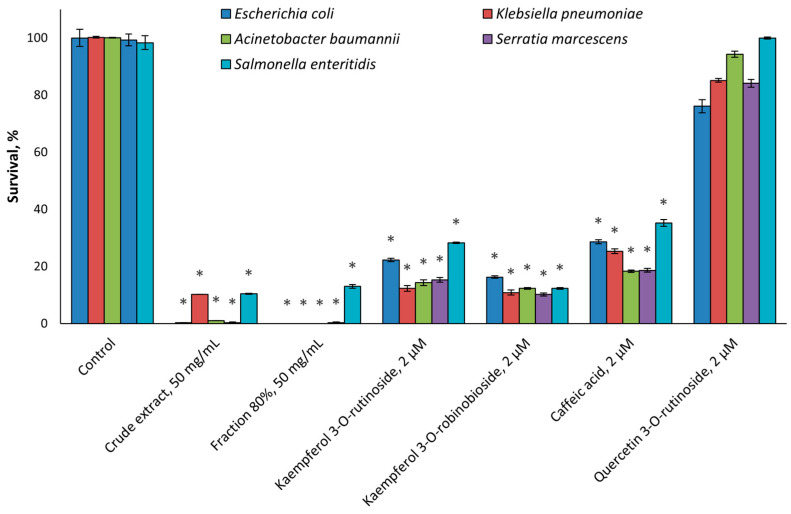
The effect of *F. benjamina* extract, active fraction, and its identified flavonoid phytochemicals on survival of the drug-resistant bacteria. The concentration of the crude extract was 50 mg/mL, and the concentration of the 80% fraction MeOH was 50 mg/mL; the identified phytochemicals were taken at concentration of 2 µM. Three independent experiments were performed. Asterisks indicate *p* < 0.001 as compared to the respective controls.

We evaluated in vitro the antimicrobial activity of the crude extract at the concentration of 50 mg/mL in PBS, its fractions at the concentration of 50 mg/mL in PBS, and the identified compounds at a concentration of 2 µM. PBS served as a negative control. In the experiment with the biofilm-producing bacterium, streptomycin served as a positive control. Still, it was not able to kill the drug-resistant *Escherichia coli*, *Klebsiella pneumoniae*, *Acinetobacter baumannii*, *Serratia marcescens*, and *Salmonella enteritidis* bacteria (Figure 1).

According to Figure 1, the leaf extract, the 80% fraction, and all components of this fraction except quercetin 3-*O*-rutinoside demonstrated significant antimicrobial activity against all the tested drug-resistant microorganisms (*p* < 0.001). The other fractions of the extract and the other identified compounds were ineffective. Moreover, the active fraction inhibited the development of *Escherichia coli*, *Klebsiella pneumoniae*, *Acinetobacter baumannii*, and *Serratia marcescens* (Figure 1).

**Figure 2:**
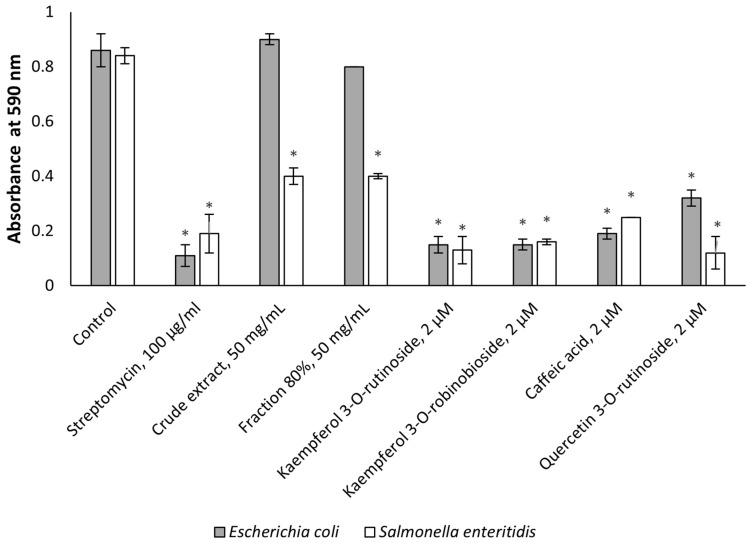
*Ficus benjamina* extract, active fraction, and flavonoid phytochemicals affect biofilm formation. The concentrations of the crude extract and the 80% fraction were 50 mg/mL; streptomycin was at a concentration of 100 µg/mL; the flavonoids were applied at a concentration of 2 µM. Three independent experiments were performed. Asterisks indicate *p* < 0.001 as compared to the respective controls.

Figure 2 shows that the effect of the crude extract and the active fraction was not significant on biofilm formation in the case of *E. coli.* Still, the identified flavonoids had substantial inhibitory activity (*p* < 0.001). In the case of *S. enteritidis*, all tested plant material was significantly (*p* < 0.001) effective. Moreover, kaempferol 3-*O*-robinobioside and quercetin 3-*O*-rutinoside acted better than streptomycin.

### 2.3. Effect on Cancer Cell Line Proliferation

**Figure 3:**
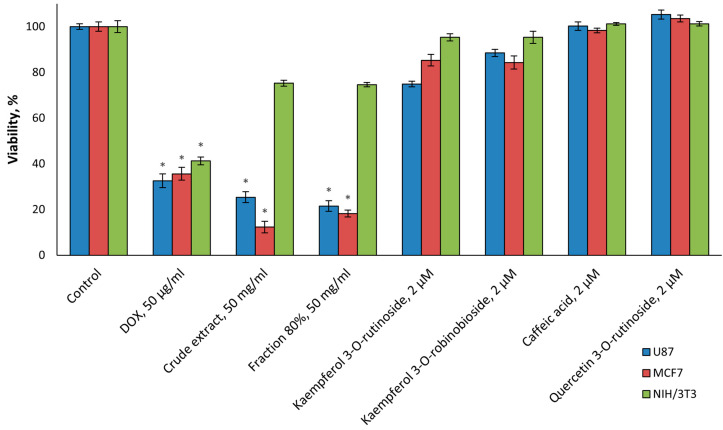
Effect on cancer cell line proliferation of *F. benjamina* extract, active fraction, and flavonoid phytochemicals. The concentrations of the crude extract and the fraction were 50 mg/mL; DOX was at a concentration of 50 µg/mL; the flavonoids were applied at a concentration of 2 µM. NIH/3T3 (noncancerous cells) and MCF7 and U87 (cancer cell lines) were used. Three independent experiments were performed. Asterisks indicate *p* < 0.001 as compared to the respective controls.

The effect on cancer cell line proliferation was investigated based on the viability of MCF7 and U87 (cancer cell lines) and NIH/3T3 (noncancerous cells). A *F. benjamina* extract and the 80% fraction significantly (*p* < 0.001) killed both cancer cell lines and were more effective than DOX, the positive control (Figure 3). The compounds of this fraction also decreased the viability of cancer cell lines, but this change was not significant (Figure 3).

### 2.4. Pro-WH Activity

**Figure 4:**
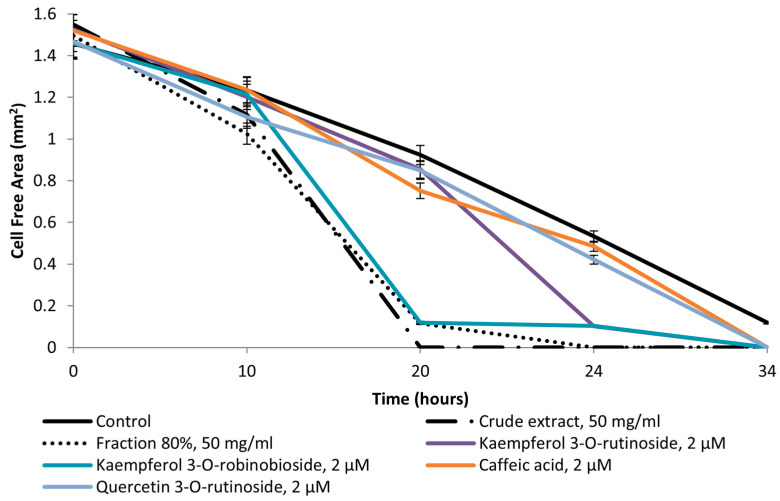
Pro-WH effect of *F. benjamina* extract, active fraction, and flavonoid phytochemicals. The rate of gap closure in cultured human dermal fibroblasts (scratch assay: in vitro model of wound healing) at 0, 10, 20, 24, and 34 h after wound generation. Untreated fibroblasts were used as control. The concentrations of the crude extract and the 80% fraction were 50 mg/mL; the flavonoids were applied at concentration of 2 µM. Three independent experiments were performed.

With regard to its pro-WH properties, Figure 4 demonstrates that the best pro-WH properties belonged to the crude extract which enhanced WH significantly (*p* < 0.001) as complete WH was observed in 20 h. Only the 80% fraction was effective (*p* < 0.001), but it acted slightly less than the extract (Figure 4). Kaempferol 3-*O*-robinobioside, a flavonoid component of the active fraction, enhanced WH significantly 20 h after the treatment (*p* < 0.001) but slightly less than the extract and the active fraction. Before these experiments, the extract’s cytotoxicity and its components were determined. The extract was not toxic for HDF cells at a concentration of less than 1000 µg/mL, for the active fraction at a concentration less than 1400 µg/mL, and for all tested compounds at a concentration of 100 µg/mL at least (the results are not presented).

### 2.5. Anti-Inflammatory Activity

**Figure 5:**
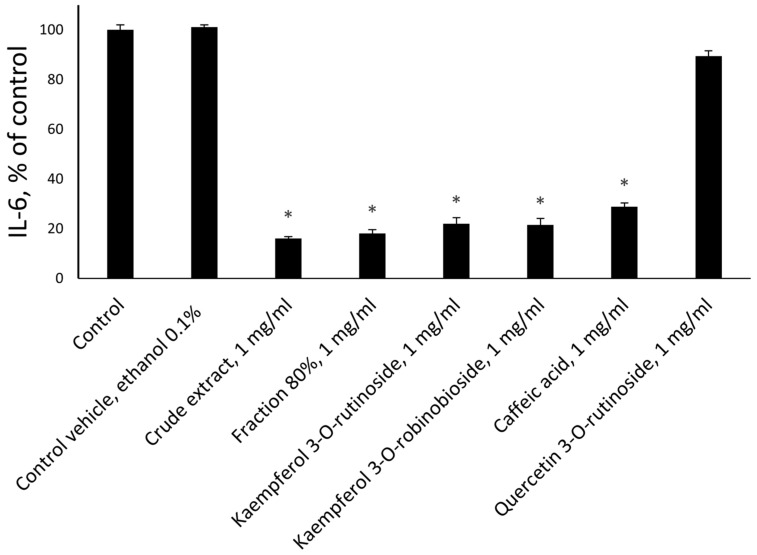
*Ficus benjamina* extracts and its flavonoid phytochemicals affect the secretion of pro-inflammatory cytokines, IL-6 from human dermal fibroblasts. The concentration of the extracts, fractions and phytochemicals was 1 mg/mL. The final ethanol concentration was 0.1%. Untreated cells were used as controls. Control vehicle treatments were used to exclude the effect of ethanol on the cells. Three independent experiments were performed. Asterisks indicate *p* < 0.001 as compared to the respective controls.

**Figure 6:**
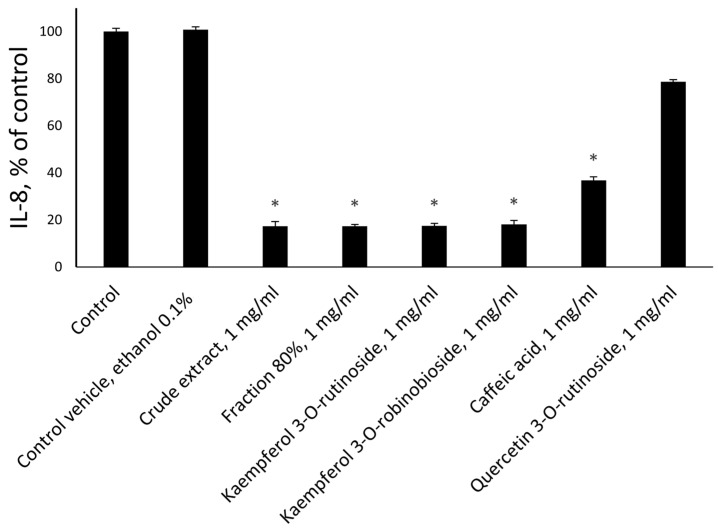
*Ficus benjamina* extracts and its flavonoid phytochemicals affect the secretion of pro-inflammatory cytokines, IL-8 from human dermal fibroblasts. The concentration of the extracts, fractions, and phytochemicals was 1 mg/mL. The final ethanol concentration was 0.1%. Untreated cells were used as controls. Control vehicle treatments were used to exclude the effect of ethanol on the cells. Three independent experiments were performed. Asterisks indicate *p* < 0.001 as compared to the respective controls.

Further experiments investigated the expression of pro-inflammatory cytokines IL-6 (Figure 5) and IL-8 (Figure 6) from the HDFs. We found that their secretion was significantly reduced after treatment by crude extract, the 80% fraction, caffeic acid, kaempferol 3-*O*-rutinoside, and kaempferol 3-*O*-robinobioside (*p* < 0.001).

All tested flavonoids were active with the exception of quercetin 3-*O*-rutinoside (Figure 5 and Figure 6). The identified volatile compound did not influence IL-6 or IL-8 production (data are not present).

### 2.6. Bioinformatic Analysis of Identified Phytochemicals

**Figure 7:**
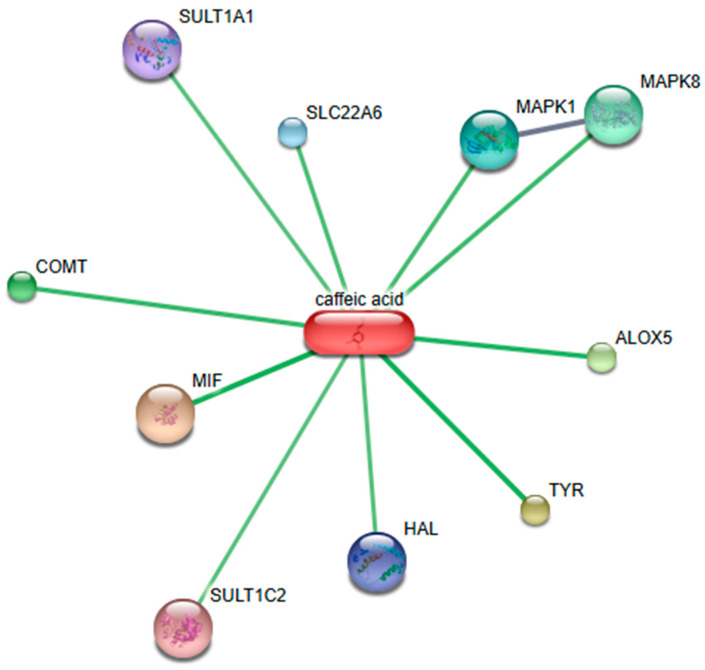
Protein–protein and protein–compound interactions between human targets of caffeic acid. This figure was constructed using the default settings of the STITCH database (http://stitch.embl.de/, accessed on 4 April 2024).

Bioinformatic analysis was performed for the elucidation of the human therapeutic targets (proteins and other biomolecules) of the compounds, identified in *F. benjamina*. This analysis demonstrated that caffeic acid had a wide variety of targets in the human organism. Figure 7 shows that interacting protein targets are strongly connected with Sulfotransferases SULT1A1 and SULT1C2, Catechol-*O*-methyltransferases (COMT), macrophage migration inhibitory factor (MIF), histidine ammonia-lyase (HAL), tyrosinase (TYR), lipoxygenase (ALOX5), some mitogen-activated protein kinases (MAPK1 and MAPK8) and the organic anion transporter 1 or solute carrier family 22 member 6 (SLC22A6).

## 3. Discussion

The thorough mechanistic research presented in this article highlights that *Ficus benjamina* is a plant with diverse biological activities and rich chemical content. We mentioned that all identified compounds (Table 1) were purchased for our follow-up experiments. In addition to those, we studied several compounds that were previously reported in the literature, including chlorogenic, *p*-coumaric, ferulic and syringic acids in roots, chlorogenic *p*-coumaric and ferulic acids in stem [3], and quercetin in leaves [21], but were not identified in our samples. This discrepancy could be explained by the fact that different chemotypes are observed among various plants of *F. benjamina* which are defined by differences in the phytochemical content of the plants of the same species both in quantity and quality [22]. In support of this notion is an observation that fruits of one of the varieties of *F. benjamina* contain six new isoflavones and 2-coumarano-chroman-4-one, along with fifteen known compounds [23].

We observed that the tested plant and many of its compounds were effective against both various drug-resistant bacteria and biofilm-forming microorganisms (Figure 1 and Figure 2). Although antimicrobial properties of *F. benjamina* were investigated previously [3], this is the first report showing the above-mentioned antimicrobial activities. With regards to the tested compounds, caffeic acid was mentioned in many publications as an antibacterial agent [24,25,26], and the mechanism of its action is associated with its ability to inhibit the efflux pumps of bacteria [26]. This compound can affect the biofilm-forming microorganisms and drug-resistant bacteria [27]. Quercetin 3-*O*-rutinoside was reported as an antibacterial agent which is also able to inhibit the biofilm-forming and drug-resistant microorganisms [28]. Kaempferol 3-*O*-rutinoside was mentioned as a component of the extract of *Chuquiraga straminea* Sandwith which was effective against methicillin-resistant *S. aureus* [29]. Up to now, Kaempferol 3-*O*-robinobioside was reported only as an antiviral agent [4], and the set of data presented in this article revealed its antibacterial effects for the first time.

We analyzed only the viability of cancer and noncancerous cells; further research is required for the assessment of metabolic activity of cancer and noncancerous cells after treatment by phytochemicals isolated from *F. benjamina.*

Effect on cancer cell line proliferation of the extract and the active fraction were better than the individual components of this fraction (Figure 3). The synergistic effect could be a plausible explanation for such results [30,31].

Further research is necessary for identifying proper combinations of compounds of effective fractions and possibly other compounds of the extract and elucidating their anticancer action mode. It should be noted that the tested volatile compounds did not have significant anticancer activity (data are not present).

Regarding the pro-wound-healing (WH) activity, all identified flavonoids improved WH 20 h after the treatment (Figure 4). It was previously reported that many flavonoids can influence the inflammatory process [32]. The antioxidant features of caffeic acid contribute to enhancing many important processes [33], for example, pro-WH and anti-inflammatory properties [34]. Kaempferol-3-*O*-rutinoside enhanced WH, promoting keratinocyte migration by means of FAK and Rac1 activation [35].

Based on bioinformatical analysis, comparison with the literature shows that our present results are in accordance with those of Garcia-Jimenez et al. [36]: the antibacterial activity against *Bacillus subtilis*, *E. coli*, *S. aureus*, *Salmonella*, and *K. pneumoniae* was connected with the action of tyrosinase on caffeic acid.

In addition, the connection of caffeic acid with 5-lipoxygenase inhibitors may explain its anti-inflammatory effect [37].

## 4. Materials and Methods

### 4.1. Preparation of Plant Material

*Ficus benjamina* plants were obtained from the nursery of Volcani Institute, Agricultural Research Organization (Bet Dagan, Israel) with voucher number: PTBG0000003898. The plants were grown in a controlled greenhouse at Ben-Gurion University of the Negev (Beer-Sheva, Israel), and the leaves of *F. benjamina* for preparing the extract and its fractions were harvested from the Ben-Gurion University greenhouse. The extract was prepared as it was reported previously [1,4,28]. Briefly, the ethanolic extract was prepared from young leaves which were collected and dried at room temperature for seven days. The leaves were then treated with the assistance of a blender in order to destroy plant tissues. The obtained mixture was placed for 48 h in ethanol in an orbital shaker incubator under the temperature of 25 °C. The next stage was centrifuging the mixture at 2000 rpm for 10 min. After the evaporation, the pellet was weighed and dissolved to the appropriate concentrations.

### 4.2. Compounds

Quercetin, quercetin 3-*O*-rutinoside, caffeic acid, kaempferol 3-*O*-rutinoside, kaempferol 3-*O*-robinobioside, chlorogenic, chlorogenic *p*-coumaric, *p*-coumaric, and ferulic and syringic acids were purchased from S.L. Moran, Jerusalem, Israel. Cyclopropyl Methylcarbinol; 2-Propanol-1,1 dimethoxy acetate; acetic acid,1- methyl-ethyl ester; and gluoctanic acid lactone were bought at Vladiko, Rehovot, Israel. Common HPLC solvents were purchased from Merck, Kenilworth, NJ, USA. Biological Industries (Kibbutz Beit HaEmek, Israel) was a supplier of Dulbecco’s modified Eagle’s medium (DMEM), fetal bovine serum (FBS), and L-glutamine.

### 4.3. Phytochemical Analysis

The volatile compounds were identified using GC/MS analysis as previously described [28,38]. Briefly, GS/MS was performed using a Varian CP 3800 GS/MS analytical system. A qualitative analysis without extracting active compounds was fulfilled with assistance of a headspace injection mode. The identification of the volatile compounds was performed on the basis of comparison of their spectrum [28].

The extract was processed with the help of a reverse-phase, RP-C18 Sepack column (Supelco, St. Louis, MO, USA) with the methanol gradient increasing from 0% (*v*/*v*) to 100% (*v*/*v*). The compounds of the active fraction, which was the 80% fraction (*v*/*v*), were identified using two analytical methods: HPLC and LC-ESI-MS [4,28]. HPLC was performed with the following characteristics: solution A included water and acetic acid (97:3 *v*/*v*), and solution B was methanol, with a flow rate of 1.0 mL/min. The UV detector was set at 360 nm with a reverse-phase column (Betasil C-18, 5 μm, 250 × 0.46 mm; Thermo-Hypersil, Paisley, UK). The same method was used for LC-ESI-MS; the flow rate was set at 1 mL/min, and the injection volume was 10 µL. The following MS conditions were chosen for optimization of the analysis: API electron spray interface, negative mode polarity, a drying gas flow of 10 L/min, a nebulizer gas pressure of 60 psi, a drying gas temperature of 335 °C, a fragmentor voltage of 0.4 V, a capillary voltage of 4451 V, and a scan range of *m*/*z* 25–1000, at 1.15 s/scan. The quantitative estimation of concentrations of the tested compounds in the plant extract was performed on the basis of comparison with the corresponding commercial standards.

### 4.4. Bacterial Strains

The following bacterial strains, *Escherichia coli*, *Klebsiella pneumoniae*, *Acinetobacter baumannii*, *Serratia marcescens*, and *Salmonella enteritidis*, from the Immunology and Infection Diseases Hospital Laboratory of Soroka University Medical Center, Beer-Sheva, Israel, were used in our experiments.

### 4.5. Investigation of Antimicrobial Activities

The effect of the extract, the fractions, and the tested compounds on the survival of the drug-resistant bacteria and on biofilm formation was investigated as we have previously described [28]. Colloquially, the bacterial colonies were placed in peptone water growing media and incubated at 35 °C for 24 h. Phosphate buffered saline (PBS) (Hylabs, Rehovot, Israel) was used for rinsing via centrifugation (6000 rpm) and resuspending.

The microorganisms were estimated and calculated using the heterotrophic plate count (HPC) at the beginning of the experiment and at the 20 h time point.

### 4.6. Investigation of Effect on Cancer Cell Line Proliferation

The following cell lines were used for experiments: MCF7 (human breast carcinoma cell line), U-87 (human glioblastoma carcinoma cells line), NIH/3T3 (*Mus musculus*, mouse embryo fibroblasts), and 3T3-L1 mouse adipocytes. All cells were purchased from the American Type Culture Collection (ATCC), Rockville, MD, USA. All tested cell lines were maintained in DMEM containing 10% FCS, 1% l-glutamine, and 1% penicillin/streptomycin/nystatin at 37 °C in a humidified atmosphere with 5% CO_2_. The viability assay was performed as described previously [39]. CellTiter-Fluor™ Cell Viability Assay was purchased from Promega (Madison, WI, USA).

### 4.7. Investigation of Pro-WH Activity

Propagation of Human Dermal Fibroblasts (HDF) was performed as described in Section 4.6. In order to estimate the toxic properties of the tested extract and its components, the neutral red cytotoxicity assay was employed [40]; the non-toxic concentrations were tested. The estimation of pro-WH activity in vitro was detailed previously [38]. Briefly, the scratch assay was handled according to the Ibidi silicone inserts’ manufacturer recommendations (Ibidi, Fitchburg, WI, USA). The experiments involve placing these inserts into 12-well assay plates, disseminating the cells along both sides of the inserts, producing wound gaps, deleting the inserts in 24 h, and the supplementation of the tested compounds in non-toxic concentrations and measurements of the cell-free area. The measurements were carried out at 0, 10, 20, 24, and 34 h after starting the experiment.

### 4.8. Investigation of Pro-Inflammatory Cytokine Secretion

The concentrations of interleukin-6 (IL-6) and interleukin-8 (IL-8) were determined with assistance of an ELISA kit (R&D Systems, Minneapolis, MN, USA). The estimation of pro-inflammatory cytokine secretion was described previously in accordance with the instructions of the manufacturer [38].

### 4.9. Bioinformatic Analysis

The STITCH database was used for bioinformatical analysis [41].

### 4.10. Data Analysis

Independent experiments were conducted three times at least. Microsoft Excel 365 (Microsoft Corporation, Redmond, WA, USA) was used for the analysis. Mean ± standard error values were compared using Student’s *t*-test.

## 5. Conclusions

All in all, the set of data presented here demonstrates that *F. benjamina* is a plant with promising medicinal properties. Our models show that it contains antimicrobial, pro-WH, anticancer, and anti-inflammatory agents. Further studies should be performed in in vivo models to elucidate this plant’s full therapeutic potential and its active compounds. Elucidation of the molecular mechanisms of action of the tested compounds also warrants further investigation and can help in exploiting this plant and its phytochemicals as novel therapeutic agents.

## Figures and Tables

**Table 1:** Phytochemicals identified in *Ficus benjamina*.

Compound	Molecular Structure	Methods of Identification	Conc., mg/kg
Caffeic acid	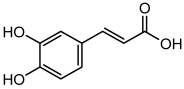	HPLC LC-ESI-MS	7.6 ± 0.58
Quercetin 3-*O*-rutinoside	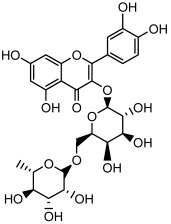	HPLC LC-ESI-MS	11.5 ± 0.21
Kaempferol 3-*O*-robinobioside	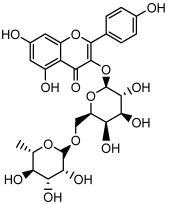	HPLC LC-ESI-MS	4.3 ± 0.19
Kaempferol 3-*O*- rutinoside	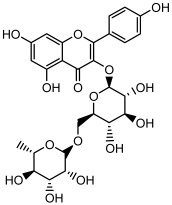	HPLC LC-ESI-MS	3.9 ± 0.55
Cyclopropyl Methylcarbinol	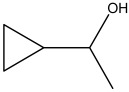	HS-GS-MS	7.11 ± 0.32
2-Propanol-1,1 dimethoxy acetate	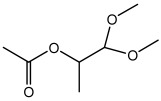	HS-GS-MS	46.91 ± 0.18
Acetic acid,1- methyl-ethyl ester	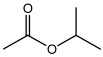	HS-GS-MS	26.33 ± 0.97
Gluoctanic acid lactone	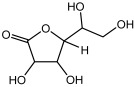	HS-GS-MS	286.63 ± 1.07

## Data Availability

The original contributions presented in this study are included in the article. Further inquiries can be directed to the corresponding authors.
